# Effect of a typical systemic hospital reform on inpatient expenditure for rural population: the Sanming model in China

**DOI:** 10.1186/s12913-019-4048-7

**Published:** 2019-04-16

**Authors:** Zhaolin Meng, Min Zhu, Yuanyi Cai, Xiaohong Cao, Huazhang Wu

**Affiliations:** Department of Health Service Management, School of Humanities and Social Sciences, China Medical University, No.77 Puhe Road, Shenyang, 110122 Liaoning China

**Keywords:** Systemic hospital reform, Inpatient expenditure, NCMS, China

## Abstract

**Background:**

Considering catastrophic health expenses in rural households with hospitalised members were unproportionally high, in 2013, China developed a model of systemic reform in Sanming by adjusting payment method, pharmaceutical system, and medical services price. The reform was expected to control the excessive growth of hospital expenditures by reducing inefficiency and waste in health system or shortening the length of stay. This study analyzed the systemic reform’s impact on the financial burden and length of stay for the rural population in Sanming.

**Methods:**

A total of 1,113,615 inpatient records for the rural population were extracted from the rural new cooperative medical scheme (NCMS) database in Sanming from 2007 to 2012 (before the reform) and from 2013 to 2016 (after the reform). We calculated the average growth rate of total inpatient expenditures and costs of different medical service categories (medications, diagnostic testing, physician services and therapeutic services) in these two periods. Generalized linear models (GLM) were employed to examine the effect of reform on out-of-pocket (OOP) expenditures and length of stay, controlling for some covariates. Furthermore, we controlled the fixed effects of the year and hospitals, and included cluster standard errors by hospital to assess the robustness of the findings in the GLM analysis.

**Results:**

The typical systemic reform decreased the average growth rate of total inpatient expenditures by 1.34%, compared with the period before the reform. The OOP expenditures as a share of total expenditures showed a downward trend after the reform (42.34% in 2013). Holding all else constant, individuals after the reform spent ¥308.42 less on OOP expenditures (*p* < 0.001) than they did before the reform. Moreover, length of stay had a decrease of 0.67 days after the reform (p < 0.001).

**Conclusions:**

These results suggested that the typical systemic hospital reform of the Sanming model had some positive effects on cost control and reducing financial burden for the rural population. Considering the OOP expenditures as a share of total expenditures was still high, China still has a long way to go to improve the benefits rural people have enjoyed from the NCMS.

**Electronic supplementary material:**

The online version of this article (10.1186/s12913-019-4048-7) contains supplementary material, which is available to authorized users.

## Background

Containing health care costs has been put high on the agenda of many low-and middle-income countries (LMICs), because health costs tend to grow faster than the economy in these resource constrained countries, leading to concerns about the sustainability of health care expenditures and heavy financial burden on vulnerable populations, including rural populations [1, 2]. Authors have documented that in LMICs, such as China, India, Malawi, and Vietnam, excessive reliance on OOP payments for the rural population leads to financial barriers or impoverishment [3–6]. Studying how to control rapid growth of health expenditures and reduce the OOP expenditures is vital to achieve the Sustainable Development Goals (SDGs) in LMICs.

By 2007 almost the entire China’s population were covered by public health insurance schemes [7]. Acting as one of the basic foundations of Chinese rural healthcare, the NCMS has played an important role in guaranteeing acquisition of medical healthcare for the rural population. By the end of 2017, NCMS had covered all counties and villages, accounting for 99.36% of the total rural population [8]. Almost all the rural population were covered by NCMS, however, this modest health insurance scheme only covered some hospital expenses for rural residents. Xie et al. [9] and Li et al. [3] found that catastrophic health expenses and medical impoverishment in rural households with hospitalised members were still unproportionally high. It is too early to claim that China has achieved universal coverage of health care as is defined by the World Health Organization (WHO) [10].

The Chinese government has committed to increasing funding for the NCMS; however, the increased funds have to be accompanied by effective cost control measures. The astronomical growth of total health expenditures has imposed great burdens to the whole country [11]. Although some pilot reforms to contain health expenditure growth have been experimented in China, few have produced significant effects [12, 13].

In 2013, Sanming city in Southern China embarked on a systemic hospital reform to control the excessive growth of hospital expenditures, which is highly recognized by the China central government [14]. However, despite the attention given to the Sanming reform model, its impact still remains unclear. He et al. [15] found that although the pharmaceutical reform in the Sanming model could control or reduce drug expenditures and total health expenditures in public hospitals in the short term, expenditures gradually resumed growing again and reached or even exceeded their baseline levels of pre-reform period. Fu et al. [14] found that the reforms in Sanming improved the performance of the public hospitals. Liu et al. [16] found that the comprehensive efficiency of general hospitals was boosted for one year after the reform of Sanming model. These studies were all about the evaluation of the reform of Sanming model at the hospital level, however, the impact of Sanming model reform has yet to be investigated at the individual level particular to the rural population. Considering medical impoverishment was more common in rural households, which are often doubly disadvantaged because their health needs are greater but their economic resources are severely constrained [17]. Ensuring that rural populations have access to affordable health care is the key to the equal access to health care. Moreover, previous studies only evaluated short-term effects about the Sanming model. This study aimed to evaluate the effect of the Sanming model systemic reform on inpatient expenditures for the rural population using 10 years of real-world insurance claims data.

### Context: the systemic hospital reform in Sanming

Sanming was a city in Fujian province, and more than 64% of the population of which were rural residents [14]. Like many other places in China, the hospitals in Sanming also suffer from distorted provider incentives. Some physician services that require time and professional skills were charged below actual costs. Meanwhile, hospitals had to survive financially by making profits from prescribing drugs and providing high-technology tests, leading to widespread over-prescription of drugs and overuse of diagnostic tests [11].

**Fig. 1:**
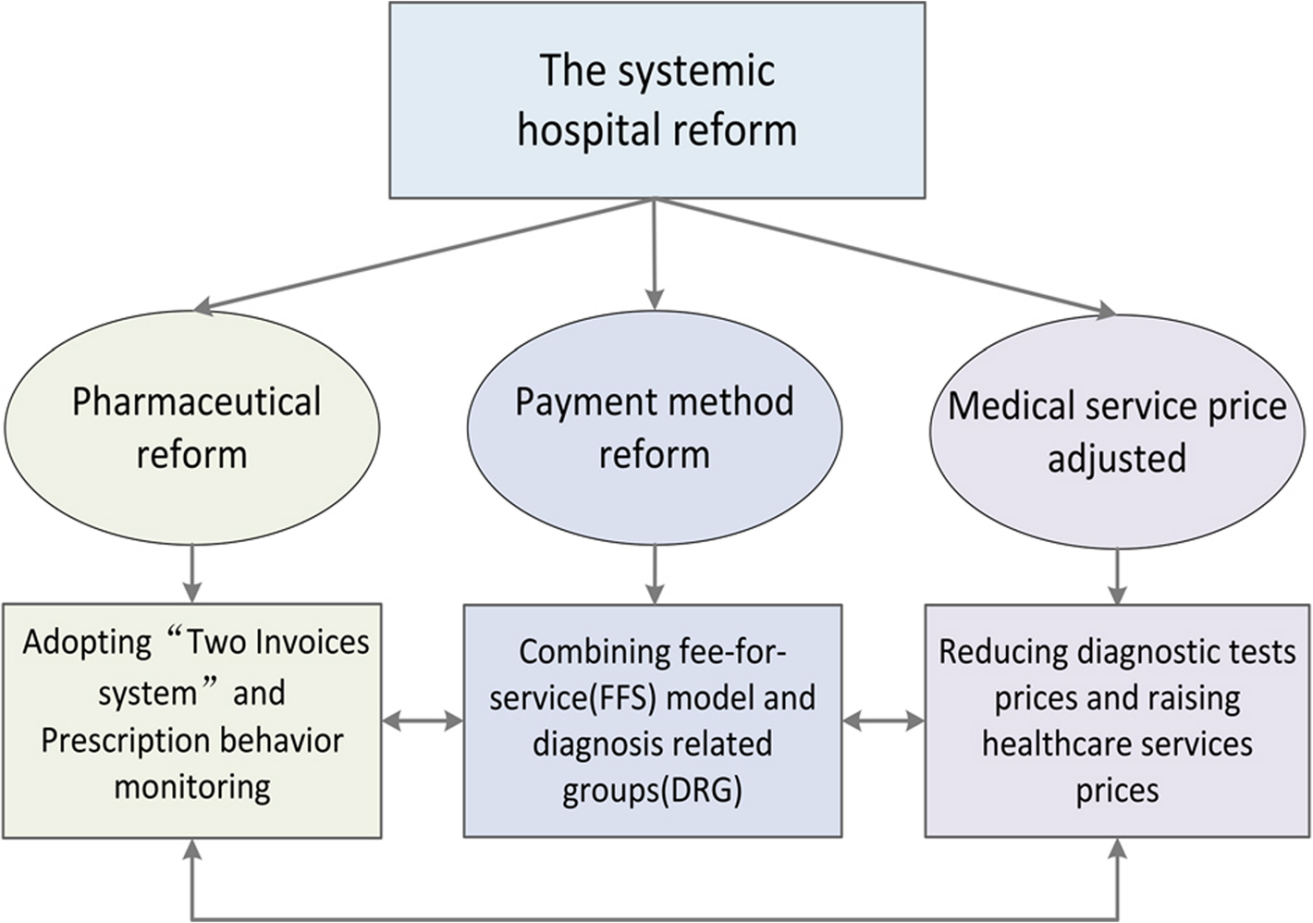
The framework of the systemic reform

In order to solve these problems, Sanming city embarked on a systemic reform in 2013. The framework of the reform was described in Fig. 1. The “Two Invoices” system was introduced to reduce unnecessary intermediate medical distributors. In Sanming, the “Two Invoices” system was only applicable to medicine, not other medical devices. Only medicines with two produced invoices, one from the manufacturer to the distributor and the other from the distributor to the hospital, can be covered by the medical insurance fund. Moreover, online monitoring of drug prescriptions was conducted to control unreasonable prescriptions. In 2014, the Development and Reform Commission, which has the power to set price, implemented a plan to modify the distorted price schedule. The prices for high-tech diagnostic tests were decreased, for example, the price of computed tomography (CT) scanning per person declined by 15%; and the government gave more subsidies to raise the prices for physician services that require time and professional skills. After these reforms, over-providing of unnecessary examinations and tests would be less profitable. And, a payment method combination of fee-for-service (FFS) model and diagnosis related groups (DRG) was implemented. The systemic hospital reform was expected to control the excessive growth of hospital expenditures by reducing inefficiency and waste in health system or shortening the length of stay.

In order to analyse how the Sanming model of systemic hospital reform affected inpatient expenditures of rural population, we retrospectively analysed a total of 1,113,615 inpatient records for the rural population. We compared the average growth rate of total inpatient expenditures and costs of different medical service categories before and after the implementation of the Sanming model of systemic hospital reform. We further investigated whether the Sanming model of systemic hospital reform affected OOP expenditures and length of stay.

## Methods

### Data source

All registered inpatient admissions for the rural population from the NCMS database in Sanming, totaling 1,113,615, from 2007 to 2016, were included in the study. The data files included a personal information file and an inpatient claims file. The personal information file includes demographic variables, such as ID, sex, age. The inpatient claims file includes diagnoses, admission and discharge dates, hospital name, hospital level and an itemized expenditure file. All expenditure variables in the article are converted to 2007 Chinese yuan (¥) using the consumer price index. Diagnoses were coded according to International Classification of Disease, 10th Revision (ICD-10). The leading cause of hospitalization used for this study was based on the chapters found in the International Shortlist for Hospital Morbidity Tabulation (ISHMT) adopted by the WHO [18].

### Study variables

We calculated total inpatient expenditures, as well as expenditures broken down by type of services including medication expenditures, diagnostic testing expenditures, physician services and therapeutic services expenditures (e.g., intravenous infusion, acupuncture, and nursing services). We also broke total inpatient expenditures down into patient OOP expenditures and government reimbursement payments. All these outcomes were calculated per individual per inpatient visit. We calculated the average growth rate (AGR) of total inpatient expenditures and the expenditure categories before the reform (2007–2012) and after the reform (2013–2016). We compared the differences in OOP expenditures and length of stay between the period before the reform (2007–2012) and that after the reform (2013–2016), controlling for some covariates. The covariates included items in the following areas:

(i) Demographic and Socio-economic status: sex, age, annual net income.
(ii) Diseases related data: chronic diseases. In this study, if one patient’s leading cause of hospitalization was diabetes mellitus, hypertensive diseases, cerebrovascular disease, renal failure, malignant neoplasms, angina pectoris, or acute myocardial infarction, the patient would be dichotomized “Chronic diseases”, others would be dichotomized “No chronic diseases” [19].
(iii) Medical institution factors: hospital level, hospital region. Hospital level was categorized into primary, secondary and tertiary. In China, tertiary hospitals have more than 500 beds, secondary hospitals have 100–499 beds, and primary hospitals have 20–99 beds [20]. Hospital regions were coded as hospitals in Sanming and hospitals in other regions.
(iv) Health insurance policy factors: the official reimbursement rate and ceiling of annual compensation per patient by NCMS for inpatient care.

### Statistical analysis

The data analysis was done using SAS version 9.3 (SAS Institute Inc., Cary, NC). Statistical significance was based on 2-sided tests and was set to 5%. The period of 10 years was divided into two groups: the period before the reform (2007–2012) and that after the reform (2013–2016) for the pooled data. We calculated the AGR of total inpatient expenditures, inpatient medication expenditures, diagnostic testing expenditures, physician services and therapeutic services expenditures in these two periods. AGR was shown as follows; a_n_ represents the value in the n year, a_0_ represents the value in the first year [21].

$$ AGR=\left(\sqrt[n]{\frac{a_n}{a_0}}-1\right)\times 100\% $$

T-test and Analysis of Variance tests were used to test the significance of differences in OOP expenditures within variables. For analysis of variance, we used post hoc multiple comparisons of Bonferroni to compare the differences between every two groups. GLM were employed to examine the effects of reform on OOP expenditures and length of stay, controlling for those covariates. The variance inflation factor for all variables used in the model was found to be < 3.5, indicating no multicollinearity problems. Considering that a large sample is likely to produce a significant result, and so it is not necessary for a statistically significant parameter to be of practical significance. The results will also be interpreted according to the practical conditions.

We carried out two tests to assess the robustness of the findings in the GLM models. The first test was to control the fixed effects of the year and hospitals, to adjust for unobserved underlying factors that change over time and unobserved hospital-specific effects. The second test was to include cluster standard errors by hospital to account for interactions among groups of patients who were admitted to the same hospital. Clustering may occur because of correlation between observations within groups, and it is recommended by researchers to concern with the presence of clustering because its effects can hinder their ability to conduct proper statistical inference [22].

## Results

### Per capita total inpatient expenditures and expenditure categories

**Fig. 2:**
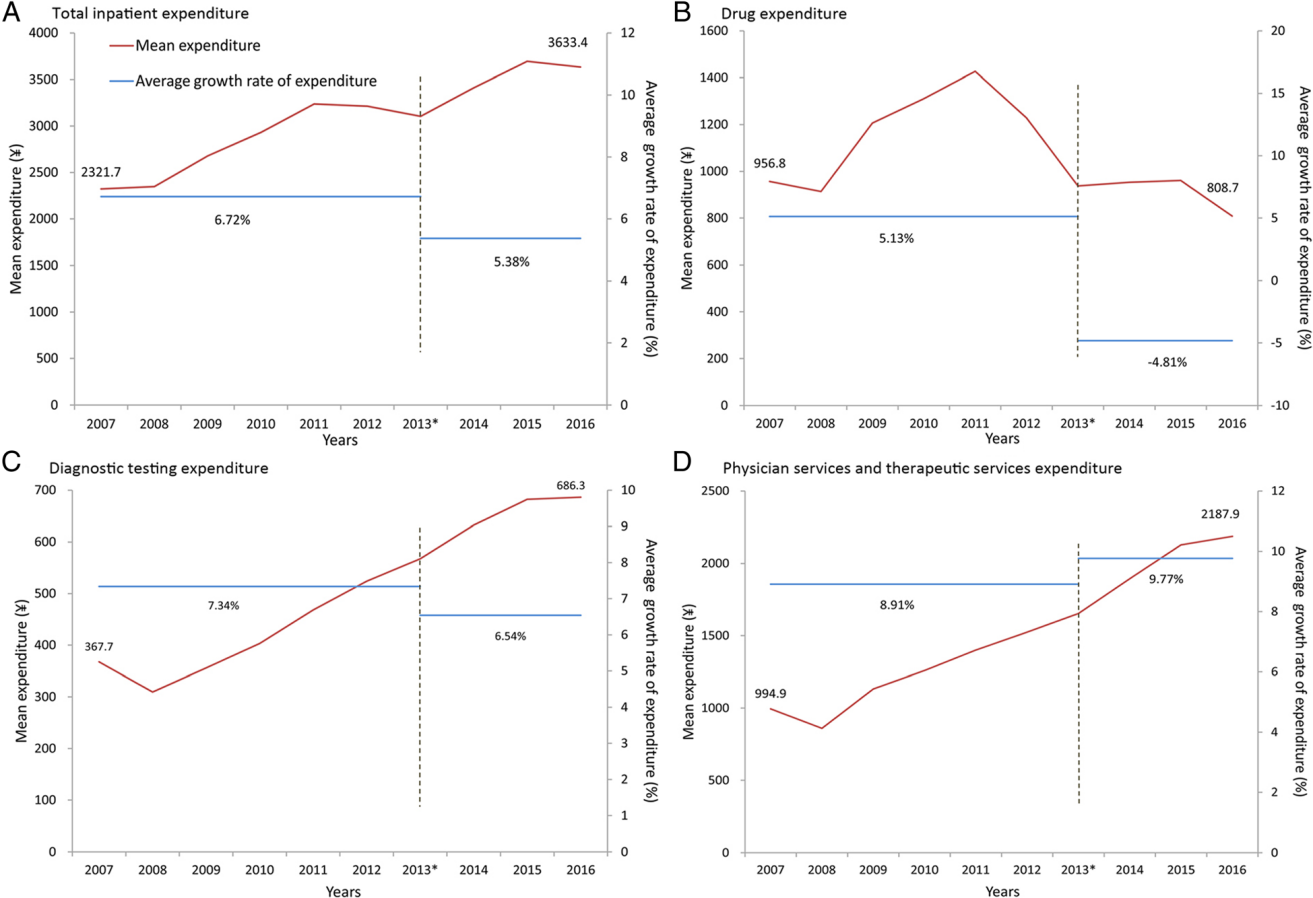
The average growth rate of total expenditures and expenditure categories before and after the reform. Note: **a** Total inpatient expenditure. **b** Drug expenditure. **c** Diagnostic testing expenditure. **d** Physician services and therapeutic services expenditure. The formula of the average growth rate (AGR) before the reform (2007–2012) was ${AGR}_{2007-2012}=\left(\sqrt[5]{\frac{Expenditure2012}{Expenditure2007}}-1\right)\times 100\%$. The formula of AGR after the reform (2013–2016) was ${AGR}_{2013-2016}=\left(\sqrt[3]{\frac{Expenditure2016}{Expenditure2013}}-1\right)\times 100\%$

As shown in Fig. 2, the average growth rate of total inpatient expenditures after the reform decreased by 1.34%, compared with that before the reform (5.38% vs 6.72%) (Panel A). The magnitude of the reduction in per capita drug expenditures is striking. The net per capita drug expenditures and the average growth rate of drug expenditures were both reduced after the reform, with the average growth rate of drug expenditures dropping from 5.13% before the reform to − 4.81% after the reform (Panel B). The diagnostic testing expenditures as a share of total expenditures had no great differences before 2013 vs. after 2013 (Panel C). The physician services and therapeutic services expenditures as a share of total expenditures, reflecting physician’s labour input, increased after the reform (Panel D).

### NCMS reimbursement policies and per capita OOP expenditures

Table 1 shows how the rural patients benefited from the reimbursement policies of NCMS in Sanming. The official reimbursement rate and the ceiling of annual compensation per patient were increased from 2007 to 2016. Table 1 also shows that on the whole, per capita OOP expenditures as a share of total expenditures and per capita OOP expenditures as a share of annual net income presented a declining trend after the reform.

**Table 1:** NCMS^a^ reimbursement policies and per capita OOP^b^ expenditures

	Year	Official reimbursement rate ^c^(%)			Ceiling of annual compensation per patient (10^4^¥)	OOP^b^ expenditure as a share of total expenditure (%)	OOP^b^ expenditure as a share of annual net income (%)
		Primary hospital	Secondary hospital	Tertiary hospital			
Before the reform	2007	65	45	35	2	67.98	30.29
	2008	75	50	35	2	59.25	25.46
	2009	80	60	40	4	58.11	25.93
	2010	80	60	40	6	57.20	25.28
	2011	85	65	45	7	53.97	22.66
	2012	60–90	80	50	8	47.60	17.54
After the reform	2013	60–95	85	50–70	10	42.37	13.33
	2014	60–95	75–85	50–80	10	47.34	13.95
	2015	60–95	75–85	50–80	10	45.10	12.68
	2016	90	85	85	10	41.05	10.58

^a^NCMS: New Cooperative Medical Scheme

^b^OOP: Out-of-pocket

^c^This is the official reimbursement rate by NCMS for inpatient care, which is not the same as the actual reimbursement ratio, because many services provided in the hospitals are not covered by NCMS, and therefore, patients have to pay 100%

### The effect of reform on per capita OOP expenditures

As shown in Table 2, per capita unadjusted OOP expenditures after the reform was significantly lower, compared with the period before the reform (¥1685.9 vs. ¥1784.1). Moreover, significant differences in per capita OOP expenditures were found by specific demographic characteristics, chronic diseases, medical institution factors, and health insurance policy factors.

**Table 2:** Distribution of OOP^a^ expenditures for rural population

	N(%)	OOP^a^ expenditure(¥)		t/F	*p*
		Mean	95%CI		
Reform category				12.32	<0.001
Before the reform(2007–2012)	429,207 (38.5)	1784.1	1771.5–1796.7		
After the reform(2013–2016)	684,408 (61.5)	1685.9	1676.6–1695.2		
Sex				−7.99	<0.001
Male	536,520 (48.2)	1755.5	1744–1767.1		
Female	577,095 (51.8)	1694.1	1684.5–1703.8		
Age, years				49.98	<0.001
< 65	749,120 (67.3)	1850.5	1841.0–1860.1		
≥ 65	364,495 (32.7)	1463.1	1451.3–1475.0		
Annual net income per capita^b^,¥				59.80	<0.001
< 7000	96,611 (8.7)	1607.6	1584.4–1630.7		
7000–14,000	589,879 (53.0)	1753.9	1743.3–1764.6		
≥ 14,000	427,125 (38.4)	1708.3	1696.6–1719.9		
Level of Hospital^b^				82,061.2	<0.001
Primary	495,267 (44.5)	766.8	758.2–775. 5		
Secondary	439,686 (39.5)	1494.8	1488.6–1500.9		
Tertiary	178,662 (16.0)	4940.8	4907.7–4973.9		
Hospital regions				− 219.04	<0.001
In Sanming	1,001,205 (89.9)	1101.8	1097.7–1105.8		
In other region	112,410 (10.1)	7270.5	7215.4–7325.6		
Chronic conditions				−33.56	<0.001
Yes	130,054 (11.7)	2191.6	2161.6–2221.6		
No	983,561 (88.3)	1661.9	1654.4–1669.4		
Official reimbursement rate,%				172.64	<0.001
< 80	411,794 (37.0)	2668.6	2653.4–2683.9		
≥ 80	701,821 (63.0)	1169.4	1161.9–1176.9		
Ceiling of annual compensation per patient,¥				11.82	<0.001
< 10^4^	684,408 (61.5)	1795.4	1781–1809.8		
≥ 10^4^	429,207 (38.5)	1693.9	1685.2–1702.7		

^a^OOP: Out-of-pocket. ^b^Using Bonferroni method, all results between every two groups are significantly different (*p*<0.001)

GLM analysis in column (1) of Table 3 also shows that without fixed effects of the year and hospitals, the per capita OOP expenditures after the reform was significantly associated with lower expenditures, compared with the period before the reform, after adjusting for relevant covariates. Holding all else constant, individuals after the reform spent ¥308.42 less on OOP expenditures (*p* < 0.001) than they did before the reform. The significant covariates of the per capita OOP expenditures included age (+ ¥292.88 for individuals aged ≥65 years), chronic diseases (+ ¥380.67 for individuals with chronic diseases), level of hospital (+ ¥1736.89 for individuals hospitalized at tertiary hospital vs. individuals hospitalized at secondary hospital or primary hospital), hospital region (+ ¥4515.74 for individuals hospitalized in other region vs. individuals hospitalized in Sanming), official reimbursement rate (+ ¥1889.99 per unit), and ceiling of annual compensation per patient (+ ¥438.23 for ceiling of annual compensation < ¥10^4^ vs. ≥¥10^4^).

**Table 3:** The GLM analysis results of OOP^a^ expenditures for rural population (*n* = 1,113,615)

Variable	OOP^a^ expenditure (1)	OOP^a^ expenditure (2)	OOP^a^ expenditure (3)
Reform (after vs. before^b^)	−308.42*** (11.24)	−347.59*** (12.73)	−347.59** (105.36)
Age (≥65 years vs. < 65 years^b^)	292.88*** (6.52)	303.63*** (6.53)	303.63*** (58.56)
Level of Hospital (tertiary vs. secondary/primary^b^)	1736.89*** (5.57)	n/a	n/a
Hospital region (in other region vs. in Sanming^b^)	4515.74*** (12.77)	n/a	n/a
Chronic diseases (yes vs. no^b^)	380.67*** (9.46)	305.51*** (9.63)	305.51*** (85.84)
Official reimbursement rate	1889.99*** (26.67)	2013.92*** (28.72)	2013.92* (990.92)
Ceiling of annual compensation per patient (¥10^4^ vs. < ¥10^4 b^)	−438.23*** (11.49)	− 335.00*** (14.06)	−335.00*** (77.84)
Intercept	− 7377.12*** (27.45)	604.92*** (87.31)	604.92 (361.24)
Yearly fixed effects	NO	YES	YES
Hospitals fixed effects	NO	YES	YES
R-Square	0.277	0.316	0.316
Prob>F	< 0.001	< 0.001	< 0.001
Clustered s.e.	NO	NO	YES

The results without year and hospital fixed effects are reported in column (1); the results with year and hospital fixed effects are reported in columns (2) and (3)

Robust standard errors are reported in parentheses in columns (1) and (2); standard errors clustered at the hospital level are reported in parentheses in column (3)

^a^*OOP* Out-of-pocket

^b^was the reference group

*p < 0.05; **p < 0.01; ***p < 0.001

Adjusted model adjusts for sex and individual annual net income

As shown in column (1) of Table 4, without fixed effects of the year and hospitals, and not including cluster standard errors by hospital, length of stay had a decrease of 0.67 days after the reform (*p* < 0.001), controlling for all the covariates.

**Table 4:** The GLM analysis results of length of stay for rural population (*n* = 1,113,615)

Variable	Length of stay (1)	Length of stay (2)	Length of stay (3)
Reform (after vs. before^a^)	−0.67*** (0.03)	−0.73*** (0.04)	− 0.73 (0.44)
Age (≥65 years vs. < 65 years^a^)	0.62*** (0.02)	0.95*** (0.02)	0.95*** (0.15)
Level of Hospital (tertiary vs. secondary/primary^a^)	2.36*** (0.02)	n/a	n/a
Hospital region (in other region vs. in Sanming^a^)	1.66*** (0.04)	n/a	n/a
Chronic diseases (yes vs. no^a^)	1.59*** (0.03)	1.64*** (0.03)	1.64*** (0.22)
Official reimbursement rate	4.62*** (0.08)	4.42*** (0.08)	4.42** (1.66)
Ceiling of annual compensation per patient (¥10^4^ vs. < ¥10^4 a^)	−0.49*** (0.03)	−0.84*** (0.04)	--0.84** (0.30)
Intercept	−1.60*** (0.08)	7.03*** (0.24)	7.03*** (0.90)
Yearly fixed effects	NO	YES	YES
Hospitals fixed effects	NO	YES	YES
R-Square	0.034	0.208	0.208
Prob>F	< 0.001	< 0.001	< 0.001
Clustered s.e.	NO	NO	YES

The results without year and hospital fixed effects are reported in column (1); the results with year and hospital fixed effects are reported in columns (2) and (3)

Robust standard errors are reported in parentheses in columns (1) and (2); standard errors clustered at the hospital level are reported in parentheses in column (3)

^a^was the reference group

*p < 0.05; **p < 0.01; ***p < 0.001

Adjusted model adjusts for sex and individual annual net income

### Robustness

As shown in column (2) and column (3) of Table 3, the results of OOP expenditures were robust. After we controlled the fixed effects of the year and hospitals, OOP expenditures also had a decrease after the reform (¥347.59, *p* < 0.001). Meanwhile, after cluster standard errors by hospital were included, the decrease of OOP expenditures was still statistically significant (¥347.59, *p* < 0.01).

With fixed effects of the year and hospitals in column (2) and column (3) of Table 4, length of stay had a slightly significant decrease if cluster standard errors by hospital were not included (0.73 days, p < 0.001); however, the decrease of length of stay was no longer significant after cluster standard errors by hospital were included (*p* > 0.05).

## Discussion

To the best of our knowledge, our study is the first to evaluate the effect of the Sanming model of systemic hospital reform on inpatient expenditures for the rural population using 10 years of real-world insurance claims data. Although in this study, a large sample use of administrative data allows study of real-world utilization patterns in an unselected population without nonresponse or recall bias problems, it should be noteworthy that a large sample easily produces significant results. So, we not only interpreted the results based on significance, but also interpreted it in light of the actual magnitude of the effect size, considering the practical conditions.

The average growth rate of total inpatient expenditures after the reform decreased by 1.34% (5.38% vs 6.72%), compared with the period before the reform. A previous study [23] identified four primary targets for cost containment: price controls, volume controls, budgeting and market oriented policies. Looking at the specific polices of the systemic reform in the Sanming model, we can find this reform refers to all these four targets, such as price controls through the “Two Invoices” system, volume controls through improving appropriateness of prescription medicines to prevent overtreatment, budgeting through health sectoral budgeting, and market oriented policies including payment reform of FFS model and DRG. The combination of all these cost containment targets in the systemic reform of the Sanming controlled its soaring hospital expenditure, which provide critical lessons for China and other countries with similar issues.

The amount of OOP expenditures after the reform showed a downward trend. Holding all else constant, individuals after the reform spent ¥308.42 less on OOP expenditures (*p* < 0.001) than before the reform. It was also shown that OOP expenditures had a similar significant decrease regardless as to whether the fixed effects of the year and hospitals or cluster standard errors by hospital were included in the model.

Without cluster standard errors according to hospital to account for intrahospital correlation, length of stay had a significant decrease after the reform (0.73 days, p < 0.001); however, the decrease of length of stay was no longer significant after cluster standard errors by hospital were included (*p* > 0.05). These results revealed that a slightly shorter length of stay was not mainly contributing to the savings of OOP expenditures. The OOP expenditures slowdown was largely accomplished through the systemic reform strategies.

The absolute amount of OOP expenditures decreased, however, OOP expenditures as a share of total expenditures after the reform was still 41.05–47.60%, higher than that in high-income countries which is generally below 30% [24]. It is important to study which are particularly likely to have more OOP expenditures to provide stronger risk protection for the particular population.

The results in the GLM analysis showed that OOP expenditures was higher for older people. Previous studies also indicated that older people experienced higher hospital expenditures [25, 26]. The high expenditures we found for older people and the resulting increased financial burden are particularly relevant today given China’s aging demographic. In 2016, people aged over 60 years accounted for 16.7% of the total population in China [8]. Our study showed that although the government had increased the health investment for rural populations in recent years, those vulnerable people, such as the elderly, were still not better protected. Hence, the health financing structure should concentrate on equity of China health financing.

The results in the GLM analysis also showed that individuals, hospitalized at high level of hospital and in other metropolitan areas, had significantly higher OOP expenditures. These two items share a common thread of reflecting more severe diseases, which usually need hospitalization at high level of hospital and in other metropolitan areas, resulting in more total inpatient spending and more OOP expenditures.

In our study, it is noteworthy that the official reimbursement rate and the ceiling of annual compensation had the opposite effect on OOP expenditures. The OOP expenditures rose as the official reimbursement rate increased, indicating that raising the official reimbursement rate alone couldn’t reduce the financial burden for the rural population. In China, the actual reimbursement rate was lower than the official reimbursement rate, because many services provided in the hospitals are not covered by NCMS and patients have to pay 100% for those uncovered services. Take 2016 as an example, the official reimbursement rate had been above 85%, however, the actual OOP expenditures as a share of total expenditures was 41.05%, implying that many medical services were still uncovered by NCMS. Compared with raising the official reimbursement rate that only covered a part of services, increasing ceiling of annual compensation per patient can better protect financial risks for the rural population. Other studies [9, 27] also showed that the insurance scheme with co-payment couldn’t protect poor people from becoming medically impoverished. This result is important for policy-making. For the public health insurance scheme, decision-makers should focus on how to provide patients with better financial risk protection and prevent medical impoverishment, while ensuring the sustainable development of the health insurance fund.

There are several limitations to this study. Firstly, although we used 10 years of data for our analysis, these findings cannot be interpreted as the long-term effect of the reform. Secondly, since we couldn’t obtain the data about the medical quality of primary hospitals, although the medical quality of secondary and tertiary hospitals as the main providers of inpatient medical services wasn’t impacted (this was not reported at the result part, see Additional file 1), it is not for certain whether the systemic reform had reduced the medical quality of primary hospitals. Thirdly, we only examined the effect of hospital reform in Sanming, therefore, the results may not be generalized to other areas. Consequently, policy makers and health care workers should treat the empirically results with caution, when applying the Sanming model in other contexts.

## Conclusions

Despite these limitations, our study provides evidence that some policy implications help to curb excessive growth of total inpatient expenditures and decrease OOP expenditures for the rural population to some extent. It is also noteworthy that the OOP expenditures as a share of total expenditures was still high and individuals who are elderly are particularly likely to have more OOP expenditures. Policy-makers would be helped by bearing these points in mind.

## Additional file

Additional file 1: The trends of six variables measuring medical quality of secondary and tertiary hospitals (PDF 911 kb)

## Abbreviations

AGR: Average growth rate
CT: Computed tomography
DRG: Diagnosis related groups
FFS: Fee-for-service
GLM: Generalized linear models
ICD-10: International Classification of Disease, 10th Revision
ISHMT: International Shortlist for Hospital Morbidity Tabulation
LMICs: Low- and middle-income countries
NCMS: New Cooperative Medical Scheme
OOP: Out-of-pocket
SDGs: Sustainable Development Goals
WHO: World Health Organization
