# Supplementary material for: Effect of a typical systemic hospital reform on inpatient expenditure for rural population: the Sanming model in China
Source: BMC Health Serv Res. 2019 Apr 16;19:231. doi: 10.1186/s12913-019-4048-7 (PMC6469113; doi:10.1186/s12913-019-4048-7)
Supplement: Supplementary file 1 — The trends of six variables measuring medical quality of secondary and tertiary hospitals (PDF 911 kb) [file 12913_2019_4048_MOESM1_ESM.pdf]

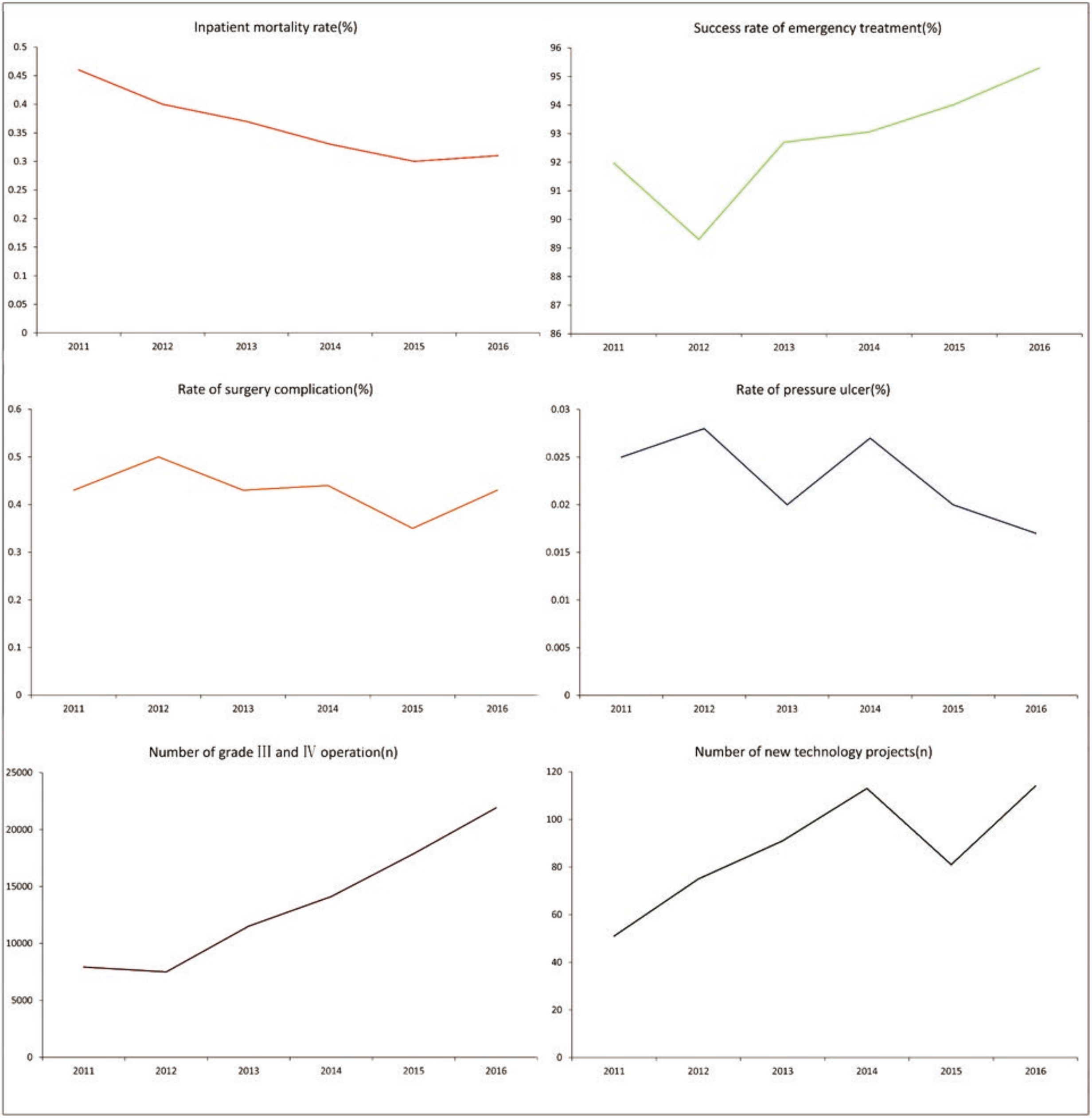

Inpatient mortality rate(%)
Success rate of emergency treatment(%)
Rate of surgery complication(%)
Rate of pressure ulcer(%)
Number of grade III and IV operation(n)
Number of new technology projects(n)
2011
2012
2013
2014
2015
2016
